# Fatal pitfalls in newborn screening for mitochondrial trifunctional protein (MTP)/long-chain 3-Hydroxyacyl-CoA dehydrogenase (LCHAD) deficiency

**DOI:** 10.1186/s13023-018-0875-6

**Published:** 2018-07-20

**Authors:** Amelie S. Lotz-Havla, Wulf Röschinger, Katharina Schiergens, Katharina Singer, Daniela Karall, Vassiliki Konstantopoulou, Saskia B. Wortmann, Esther M. Maier

**Affiliations:** 1 Department of Inborn Errors of Metabolism, Dr. von Hauner Children’s Hospital, Ludwig-Maximilians-University, Lindwurmstr. 4, 80337 Munich, Germany; 2 Becker and colleagues laboratory, Fuehrichstr. 70, 81671 Munich, Germany; 3 Clinic for Pediatrics, Inherited Metabolic Disorders, Medical University of Innsbruck, Innsbruck, Austria; 4 Department of Paediatrics and Adolescent Medicine, Medical University of Vienna, Vienna, Austria; 5 Department of Pediatrics, Paracelsus Medical University Salzburg, Muellner Hauptstr. 48, 5020 Salzburg, Austria

**Keywords:** LCHAD, Missed cases, MTP, Newborn screening

## Abstract

**Background:**

Mitochondrial trifunctional protein (MTP) and long-chain 3-hydroxyacyl-CoA dehydrogenase (LCHAD) deficiency are long-chain fatty acid oxidation disorders with particularly high morbidity and mortality. Outcome can be favorable if diagnosed in time, prompting the implementation in newborn screening programs. Sporadic cases missed by the initial screening sample have been reported. However, little is known on pitfalls during confirmatory testing resulting in fatal misconception of the diagnosis.

**Results:**

We report a series of three patients with MTP and LCHAD deficiency, in whom diagnosis was missed by newborn screening, resulting in life-threatening metabolic decompensations within the first half year of life. Two of the patients showed elevated concentrations of primary markers C16-OH and C18:1-OH but were missed by confirmatory testing performed by the maternity clinic. A metabolic center was not consulted. Confirmatory testing consisted of analyses of acylcarnitines in blood and organic acids in urine, the finding of normal excretion of organic acids led to rejection and underestimation of the diagnosis, respectively. The third patient, a preterm infant, was not identified in the initial screening sample due to only moderate elevations of C16-OH and C18:1-OH and normal secondary markers and analyte ratios.

**Conclusion:**

Our observations highlight limitations of newborn screening for MTP/LCHAD deficiency. They confirm that analyses of acylcarnitines in blood and organic acids in urine alone are not suitable for confirmatory testing and molecular or functional analysis is crucial in diagnosing MTP/LCHAD deficiency. Mild elevations of primary biomarkers in premature infants need to trigger confirmatory testing. Our report underscores the essential role of specialized centers in confirming or ruling out diagnoses in suspicious screening results.

## Background

Deficiencies of the mitochondrial trifunctional protein (MTP) (OMIM# 609015) are autosomal recessively inherited disorders of long-chain fatty acid oxidation with an estimated frequency of 1:140,000 [1–3]. MTP is an octameric multienzyme complex, which is composed of 4 alpha and 4 beta subunits encoded by the *HADHA* and *HADHB* gene, respectively. It harbors three enzyme activities: long-chain enoyl-CoA hydratase, long-chain 3-hydroxyacyl-CoA dehydrogenase (LCHAD), and long-chain 3-ketoacyl-CoA thiolase (LCKAT) catalyzing the last three steps of long-chain fatty acid oxidation [4–7]. General MTP deficiency is characterized by reduced activities of all three MTP enzymes [1]. The most common defect of the MTP complex, however, is isolated LCHAD deficiency (LCHADD) (OMIM# 609016), which is defined by reduced LCHAD activity with substantial preservation of the other two MTP enzyme activities [2]. Both MTP deficiency and isolated LCHADD lead to an accumulation of toxic ß-oxidation intermediates causing acute symptoms as well as long-term complications. Clinical symptoms mainly develop during periods of illness or fasting and affect organs preferring long-chain fat as primary source of energy, such as heart and skeletal muscle. Encephalopathy, hypoketotic hypoglycemia, lactic acidosis, and liver dysfunction are mostly found. Long-term complications comprise recurrent episodes of metabolic derangement, recurrent episodes of rhabdomyolysis, cardiomyopathy, feeding difficulties, peripheral neuropathy, and retinopathy [8–10].

Untreated, MTP and LCHAD deficiency is associated with particularly high morbidity and mortality [8, 10]. Therapy aims to prevent catabolic episodes inducing endogenous long-chain fatty acid oxidation as well as to restrict the intake of exogenous long-chain fatty acids [11, 12]. Further treatment options such as anaplerotic therapy with heptanoate are under evaluation [13]. Treated in time, outcome of MTP/LCHAD deficiency can be favorable [9, 14], prompting the inclusion of MTP and LCHAD deficiency into newborn screening programs [15, 16]. Method of choice is the determination of acylcarnitines by tandem mass spectrometry (MS/MS) with 3-hydroxypalmitoylcarnitine (C16-OH) and 3-hydroxyoleoylcarnitine (C18:1-OH) as primary biomarkers [17]. Of note, by MS/MS the different defects of MTP cannot be distinguished [18].

The finding of a positive newborn screening result is no definitive diagnosis. It is an indication of a child at risk and triggers confirmatory testing. Approaches for confirmatory testing may vary between centers. They include second screening in dried blood spot, analysis of acylcarnitines in plasma, and determination of organic acids in urine. Subsequently, enzyme analysis in fibroblasts, and genetic analysis are performed in different centers [16, 18, 19]. Confirmatory testing for long-chain fatty acid disorders has proven to be challenging. For very-long-chain acyl-CoA dehydrogenase deficiency (VLCADD), the most common long-chain fatty oxidation defect, it has been postulated that reliable diagnosis by MS/MS newborn screening may not be possible [20]. Missed cases due to normal or only slightly elevated VLCADD-specific biomarkers have been described [20–25] resulting in the recommendation of prompt genetic or enzymatic testing whenever VLCADD is suspected [16]. Furthermore, acylcarnitine profiles from healthy newborns during postnatal catabolism inducing fatty acid oxidation can indistinguishably imitate acylcarnitine profiles of VLCADD patients resulting in a well-known high incidence of false-positive cases in newborn screening [20].

In contrast to VLCADD, no significant rate of false-positive or false-negative cases seems to occur in screening for MTP/LCHAD deficiency [18]. Sporadic cases missed by the initial newborn screening sample have been reported [9, 19, 26]. The general performance of newborn screening regarding sensitivity, specificity, and positive predictive value has been reported for several programs with false-negative and false-positive cases leading to a modification of analyte cut-off values, the implementation of analyte ratios, or second-tier strategies [20, 25]. However, little is known on pitfalls during confirmatory testing resulting in fatal misconception of the diagnosis.

We report a series of three missed cases with MTP and LCHAD deficiency. One case was missed by misinterpreting the initial screening sample due to prematurity. Two cases were missed by inappropriate management of confirmatory testing and the failure to refer the patients to a specialized metabolic center. We wish to raise awareness that MTP/LCHAD deficiency may be missed in newborn screening and to underscore the essential role of specialized metabolic centers in confirming or rejecting the diagnosis in suspicious screening results.

## Methods

### Patients

We retrospectively collected data of 3 living patients with MTP or LCHAD deficiency cared for in a German (Munich; patient 1 and 2) and an Austrian (Salzburg; patient 3) metabolic center. Data of all three patients were reviewed at their respective metabolic center and are reported anonymized. The study is in accordance with the guidelines of the local ethical committees. The parents of all three subjects provided written informed consent.

### Biomarker analysis

Analysis of acylcarnitines was performed as part of the newborn screening program in dried blood spots. Blood samples were collected by the maternity clinic on filter paper as recommended between day 2 and day 3. MS/MS was performed as described previously [27]. In accordance with regulatory guidelines in Germany, positive screening results were reported to the sender (i.e. the maternity clinic) who communicated the results to the parents. The senders were explicitly advised by the screening laboratory to seek advice from metabolic experts about timely confirmatory testing and medical care [28].

For analysis of acylcarnitines in plasma, blood samples were collected in tubes prepared with lithium-heparin. Plasma was separated by centrifugation within one hour after sampling. Samples were stored at 4 °C until analysis. Analysis was performed using tandem mass spectrometry.

Organic acids were analyzed in urine using capillary gas chromatography followed by mass spectrometry as described before [29].

### Functional and molecular analyses

Enzyme activity was measured in cultured fibroblasts harvested by skin biopsy. Enzyme assay for LCHAD and LCKAT was performed by a standardized procedure as described before [30, 31]. Molecular analysis of the encoding *HADHA* (GenBank: NM_000182) and *HADHB* (GenBank: NM_000183) genes were performed by Sanger sequencing [8].

## Results

### Clinical findings

Clinical data of the three patients are summarized in Table 1, metabolic and genetic data in Table 2.

**Table 1:** Summary of clinical and laboratory findings of the patients

	Patient 1	Patient 2	Patient 3
Relevant medical history			
Pregnancy	HELLP	uneventful	HELLP
Gestational age	35	39	29
Features of metabolic decompensation			
Age (months)	4	8	5
Hypoglycemia	+	–	–
Lactic acidosis	+	–	+
Hyperammonemia	+	–	n. a.
Hepatopathy	+	–	+
Cardiomyopathy	+	+	+
Rhabdomyolysis	–	–	+
Long term complications			
Cardiomyopathy	–	–	–
Rhabdomyolysis	–	–	+
Retinopathy	+	–	+

Symbols and abbreviations are as follows: n. a. = not available, + = present, − = not present, HELLP: hemolysis elevated liver enzymes low platelet count

**Table 2:** Biomarkers and genetic data of the patients

Age	Acylcarnitines [μmol/l]				Dicarboxylic Acids in Urine	Molecular Genetics
	C16-OH (^c^)		C18:1-OH (^c^)			
Patient 1						*HADHA* gene: c.1528G > C (p.E510Q), homozygous
2 days^a^	1.62	(< 0.05)	0.46	(< 0.04)		
11 days^a^	0.49	(< 0.05)	0.29	(< 0.04)	normal	
4 months^b^	15.41	(< 0.17)	10.67	(< 0.10)	elevated	
Patient 2						*HADHB* gene: c.1198G > T (p.E400X)/c.442 + 663A > G, compound heterozygous
2 days^a^	0.35	(< 0.05)	0.06	(< 0.04)		
7 days^a^	0.44	(< 0.05)	0.08	(< 0.04)	normal	
8 months^b^	0.045	(< 0.01)	0.055	(< 0.01)	elevated	
Patient 3						*HADHA* gene: c.1528G > C (p.E510Q), homozygous
5 days^a^	0.31	(< 0.12)	0.47	(< 0.16)	n.a.	
5 months^b^	0.98	(< 0.10)	0.74	(< 0.10)	elevated	

Symbols and abbreviations are as follows: ^a^ filter paper, ^b^ plasma, collected in metabolic decompensation, ^c^cut-off, n.a. = not available

Patient 1, a boy, was born after 35 weeks of gestation by Caesarian section due to maternal HELLP (haemolysis, elevated liver enzymes, low platelet count) syndrome (birth weight 2120 g). Postnatal adaptation was uneventful. He is the second child of healthy, non-consanguineous parents of Caucasian origin (Germany/Romania). His older brother is healthy. Two former pregnancies of the mother ended in abortions (G4P2).

Newborn screening was performed on day 2, showing elevated concentrations of C16-OH (1.62 μmol/l; cut-off < 0.05 μmol/l) and C18:1-OH (0.46 μmol/l; cut-off < 0.04 μmol/l). A second screening specimen collected on day 11 again showed elevated C16-OH (0.49 μmol/l; cut-off < 0.05 μmol/l) and C18:1-OH (0.29 μmol/l; cut-off < 0.04) compatible with MTP/LCHAD deficiency (Table 2). Additionally, an analysis of organic acids in urine was initiated revealing normal results. Based on the normal excretion of organic acids, the diagnosis of MTP/LCHAD deficiency was considered excluded, and the patient was discharged home from the maternity clinic. A metabolic center had not been contacted.

At the age of 4 months, after an episode of vomiting and reduced food intake, the patient suffered a life-threatening metabolic decompensation. He presented with hypoglycemia, lactic acidosis (lactate 5 mmol/l; reference < 2.1 mmol/l), hyperammonemia (188 μmol/l; reference < 55 μmol/l), hepatopathy (aspartate transaminase 176 U/l, reference < 70 U/l; alanine transaminase 141 U/l, reference < 49 U/l; lactate dehydrogenase 1101 U/l, reference < 570 U/l), and severe hypertrophic cardiomyopathy (N-terminal brain natriuretic peptide 13,445 ng/l; reference < 84 ng/l). He was comatose when admitted to the emergency department of a metabolic center. The patient’s mother recalled the suspicious newborn screening result enabling prompt anabolic treatment including the restriction of long-chain fatty acids. However, the cardiovascular situation initially worsened to cardiogenic shock requiring invasive ventilation and catecholamine treatment. He recovered slowly after treatment in an intensive care unit for more than three weeks. A percutaneous endoscopic gastrostomy (PEG) tube was inserted for continuous feeding at night time and during catabolic situations. In addition, a vascular access port system was implanted due to difficult peripheral venous access.

At the time of metabolic decompensation, analysis of acylcarnitines in plasma again revealed a pattern consistent with MTP/LCHAD deficiency with elevated concentrations of C16-OH (15.41 μmol/l; cut-off < 0.17 μmol/l) and C18:1-OH (10.67 μmol/l; cut-off < 0.1 μmol/l) (Table 2). Urine analysis now showed elevated excretion of dicarboxylic acids. Diagnosis of LCHAD deficiency was confirmed by analysis of enzyme activity in fibroblasts revealing a reduced activity of LCHAD (4 nmol/min x mg protein; reference 34–114 nmol/min x mg) and a normal activity of LCKAT. Sequencing of the *HADHA* gene identified the prevalent mutation, c.1528G > C (p.E510Q), in a homozygous state (Table 2) [32–34] .

Patient 1 is now 7 years of age attending regular school. He is on a fat-modified diet strictly reduced in natural (long-chain) fat, enriched with medium-chain triglycerides (MCT), and supplemented with essential fatty acids. He receives continuous feeding during nights over a PEG tube. It was possible to terminate oral medication for cardiac insufficiency one year after metabolic decompensation. As a long-term complication of LCHAD deficiency, he has been diagnosed with early-stage retinopathy.

Patient 2, a boy, was born after 39 weeks of gestation (birth weight 2750 g). Pregnancy and postnatal adaptation were uneventful. He is the third child of healthy, non-consanguineous parents descending from Afghanistan. His older brother and sister are healthy.

Newborn screening was performed on day 2, showing elevated concentrations of C16-OH (0.35 μmol/l; cut-off < 0.05 μmol/l) and C18:1-OH (0.06 μmol/l; cut-off < 0.04 μmol/l) suspicious for MTP/LCHAD deficiency. A second screening sample collected on day 7 confirmed the elevations of C16-OH (0.44 μmol/l; cut-off < 0.05 μmol/l) and C18:1-OH (0.08 μmol/l; cut-off < 0.04 μmol/l). Analysis of organic acids in urine showed normal results (Table 2). Despite the recommendations of the newborn screening laboratory, patient 2 was not referred to a metabolic center, and dietary treatment was not initiated. The parents were informed by the maternity clinic to ensure regular food intake and to present to the primary care doctor at an early stage in case of intercurrent illness. At the age of 2 months, patient 2 was seen at the maternity clinic for clinical and biochemical follow-up. At that time, patient 2 was in good health. The acylcarnitine pattern was again suspicious for MTP/LCHAD deficiency, analysis of organic acids in urine showed an elevated excretion of dicarboxylic acids. Patient 2 was not referred to a metabolic center but was followed by the maternity clinic. At 4 months of age, echocardiography showed symmetric hypertrophy of the cardiac septum. Dietary restriction of long-chain fatty acids and supplementation of medium chain triglycerides (MCT) were initiated. Compliance to treatment was poor. No contact to a metabolic center was made. At 8 months of age, patient 2 presented with progressive dilatative cardiomyopathy and clinical signs of heart insufficiency. Eventually, he was transferred to a metabolic center. Adequate treatment was started immediately with continuous feeding via PEG tube, strict reduction of long-chain fatty acids intake, supplementation of MCT, and oral medications for cardiac insufficiency. Under this treatment, patient 2 improved, and his cardiac function fully recovered.

At the time of first admission to a metabolic center, the concentrations of C16-OH (0.045 μmol/l; reference < 0.01 μmol/l) and C18:1-OH (0.055 μmol/l; reference < 0.01 μmol/l) in plasma were elevated. Molecular analysis of the *HADHB* gene revealed two novel mutations (c.1198G > T/c.442 + 663A > G) in a heterozygous state (Table 2). Mutation c.1198G > T (p.E400X) is predicted to result in a premature stop and is absent in the Exome Aggragation Consortium (ExAC, accessed 20 Feb 2018). The transition c.442 + 663A > G in intron 7 has been postulated to show a similar pathogenic mechanism as the described transition c.422 + 614A > G. This transition has been shown to result in a cryptic splice donor spot with insertion of intronic sequences and a premature stop codon [35, 36]. In combination, these variants most likely will lead to nonsense mediated mRNA decay and finally absence of protein. Testing the patient’s parents for the identified *HADHB* mutations in order to prove the heteroallelic state was not covered by the health care system due to the refugee status of the family. To our knowledge, however, there are no reports of symptomatic MTP heterozygotes. Furthermore, molecular analysis of the *HADHA* gene did not reveal any mutations. Given the clinical and biochemical phenotype of the patient we strongly assume the mutations identified in the *HADHB* gene to be heteroallelic, thus confirming the diagnosis of MTP deficiency.

The patient is now 4 years of age. He is on a fat-modified diet and receives continuous feeding during nights over a PEG tube. It was possible to terminate oral medication for cardiac insufficiency one year after initial decompensation. So far, he experienced no long-term complications from MTP deficiency.

Patient 3, a boy, is the third child of healthy, non-consanguineous parents of Caucasian origin (Austria). His older brother and sister are healthy. One former pregnancy of the mother ended in an abortion (G4P3). He was born after 29 weeks of gestation by urgent Caesarian section due to maternal HELLP syndrome (birth weight 1040 g). He required non-invasive ventilation for 3 days (FiO_2_ 0.21) due to respiratory distress syndrome and received analeptic treatment with caffeine until day 19 of life. Parenteral nutrition was administered for 8 days. In addition, age-appropriate enteral nutrition was introduced.

Newborn screening was performed on day 5 and considered normal. Retrospective review revealed that concentrations of C16-OH (0.31 μmol/l; cut-off < 0.12 μmol/l) and C18:1-OH (0.47 μmol/l; cut-off < 0.10 μmol/l) were moderately elevated (Table 2) but did not prompt further diagnostic work-up due to normal secondary markers and normal analyte ratios. A second screening specimen after 14 days of life, recommended for infants prior to 32 weeks of gestation, was not obtained for unknown reasons.

At 5 months of age, patient 3 suffered a severe metabolic decompensation after an episode of vomiting and reduced food intake. He presented with mild lactic acidosis (3.6 mmol/l; reference < 2.1 mmo/l), hepatopathy (aspartate transaminase 614 U/l, reference 10–50 U/L; alanine transaminase 266 U/l, reference 6–53 U/l; partial thromboplastin time 178 s, reference 28–43 sec; prothrombin time 47%, reference 70–130%; fibrinogen 83 mg/dl, reference 150–380 mg/dl; antithrombin III 51%, reference 84–124%), rhabdomyolysis (creatine kinase 485 U/l; reference 41–330 U/l) and dilated cardiomyopathy (N-terminal brain natriuretic peptide 5468 ng/l; reference < 84 ng/l). The patient was admitted to a metabolic center. Biomarker analysis showed elevated concentrations of C16-OH (0.98 μmol/l; cut-off < 0.10 μmol/l) and C18:1-OH (0.74 μmol/l; cut-off < 0.10 μmol/l) in plasma (Table 2) as well as elevated excretion of dicarboxylic acids in urine. Anabolic treatment including MCT supplementation, strict restriction of long-chain fatty acids, as well as oral medications for cardiac insufficiency were initiated immediately. Under this treatment, the patient fully recovered.

Diagnosis of LCHAD deficiency was confirmed by molecular analysis of the *HADHA* gene revealing the prevalent mutation, c.1528G > C (p.E510Q), in a homozygous state (Table 2) [32–34]. Patient 3 is now 9 years of age. He is on a fat-modified diet and receives continuous feeding during nights over a PEG tube. It was possible to terminate oral medication for cardiac insufficiency one year after initial decompensation. Despite good compliance to treatment, he suffered from multiple episodes of rhabdomyolysis. In addition, he has been diagnosed with retinopathy. This patient was previously described by Karall et al. [9].

## Discussion

The performance of MS/MS screening programs and screening for single disorders including missed cases have been reported [18, 20, 25, 37]. However, little is known about pitfalls during confirmatory testing resulting in fatal misconception of the diagnosis.

We describe three missed cases in newborn screening for MTP/LCHAD deficiency, two of whom were missed during confirmatory diagnostic work-up. All three patients experienced severe metabolic decompensation, two of them life-threatening.

Patient 1 and 2 showed newborn screening results which were clearly indicative for MTP/LCHAD deficiency. Confirmatory testing was initiated by the maternity clinic and consisted of repeat analysis of acylcarnitines in dried blood spots and the determination of organic acids in urine on day 11 and day 7 of life, respectively. Despite abnormal acylcarnitines, the finding of a normal excretion of organic acids led to a rejection of MTP/LCHAD deficiency in patient 1 and an underestimation of the diagnosis and its clinical consequences in patient 2. However, most patients with fatty acid disorders show normal excretion of organic acids when well. The MTP/LCHAD-characteristic excretion of 3-hydroxydicarboxylic acids without or only small ketonuria is mostly seen during fasting and illness. False-negative results in patients with LCHADD have been reported [10]. Nevertheless, the determination of acylcarnitines in blood and organic acids in urine was recommended as initial follow-up analyses by newborn screening programs in the past [16, 38]. Current guidelines recommend prompt functional or molecular analyses as first line MTP/LCHAD deficiency confirmation exclusive of further biochemical parameters [3, 39]. LCHADD is the most common defect in disorders of the MTP complex [18] and is associated with the prevalent mutation c.1528G > C (p.E510Q) in the *HADHA* gene affecting up to 91% of defective alleles [8, 10, 32, 33]. Thus, molecular analysis is the method of choice. Enzyme testing is performed only in single laboratories, and fibroblasts are generally required. It is therefore less feasible, but may be essential in establishing the diagnosis in patients with equivocal genetic results, i.e. novel mutations and heterozygotes.

The responsible physicians in the maternity clinic of both patient 1 and 2 decided against referring the patients to a metabolic center or at least seeking metabolic specialists’ advice. Hence, they did not initiate the appropriate confirmatory testing and, moreover, misinterpreted the results in patients with a high suspicion of a condition with a considerable risk of severe decompensation. Though not mandatory, referral of newborns with suspicious screening results, or consulting a metabolic specialist to discuss the screening results and actions to be taken, is explicitly recommended in newborn screening guidelines [3, 16, 28]. Notably, for LCHADD it has been reported that immediate metabolic advice is crucial for the survival of newborns detected by newborn screening. The majority of deaths occurs before or within 3 weeks of diagnosis, especially in patients with limited approach to metabolic expertise [10]. Our report underscores the essential role of specialized metabolic centers in confirming or rejecting the diagnosis in suspicious screening results.

In patient 3, the moderately elevated concentrations of C16-OH and C18:1-OH in the initial newborn screening sample were regarded normal, presumably due to the patient’s prematurity [9]. The history of patient 3 adds to the discussion whether cut-off ranges in newborn screening may be applied for term and preterm newborns. Whereas previous data suggested that equal cut-offs may be applied [40, 41], it recently has been shown that concentrations of acylcarnitines were lower in healthy preterm compared to term newborns [42]. Many reasons have been postulated, including endogenous and metabolic differences due to lower birth weight and immaturity, and the effect of treatments administered in newborn intensive care units [42]. A nearly missed case of VLCADD probably due to dextrose infusion masking the characteristic primary markers has been reported [24]. Considering this, the moderately elevated biomarkers in preterm patient 3 may have been a consequence of increased anabolism. Our observation underlines the limitations when performing and evaluating newborn screening in preterm populations. Further studies are necessary to evaluate the need for different cut-offs in these newborns to reduce the risk of false-negative results. Moreover, the routine analysis of a second screening specimen at a later point in time, as required for infants born prior to 32 weeks of gestation in most screening programs in the United States and Europe including Austria [19, 28, 39], might have added to reduce the risk of missing the diagnosis in patient 3 [24]. The reason why no second screening specimen was obtained from patient 3 remained unclear.

Otherwise, the medical history of prematurity in combination with maternal HELLP syndrome could have facilitated the diagnosis in patient 1 and patient 3. In patients with MTP/LCHAD deficiency a remarkably high incidence of prematurity (> 60%) and maternal HELLP syndrome (approximately 25%) is found [8, 9, 14, 18, 43–45]. As a consequence, the finding of even mildly elevated biomarkers indicative for MTP/LCHAD deficiency in a premature newborn needs to prompt confirmatory testing.

Confirmatory testing of a suspicious newborn screening result does not only aim to prove the diagnosis but likewise aims to rule out the screened condition in a newborn. This applies especially for severe, unpredictable disorders with high morbidity and a remarkable mortality up to 90% like MTP/LCHAD deficiency [33].

The review of newborn screening often focusses on reliable diagnosis of relevant disease without affecting healthy individuals. It is well acknowledged that false-positive screening results do impose an unnecessary and high burden on health care systems and families. False-positive screening results have been associated with parental anxiety, stress, depression, altered parent-child relationship, and persistent misconception of ill health in the child who required follow-up testing [46, 47]. However, the experience of a life-threatening decompensation due to a formerly suspected disorder that was missed by insufficient confirmatory testing or misconception of the diagnostic work-up, severely traumatizes parents and might flaw the reputation of newborn screening.

## Conclusions

MTP/LCHAD deficiency has been implemented in many newborn screening programs. We describe two cases of LCHADD and one of MTP deficiency which were missed by either newborn screening or confirmatory testing. Two patients experienced life-threatening decompensations, one progressive cardiomyopathy. Our report highlights that the analyses of acylcarnitines in blood and urinary organic acids alone are not sufficient for confirmatory testing in MTP/LCHAD deficiency. Molecular or functional analysis is mandatory to confirm or rule out MTP/LCHAD deficiency. Even mild elevations of primary biomarkers in premature infants need to trigger confirmatory testing. Taking into account prematurity and maternal HELLP syndrome as aspects of medical history might facilitate the evaluation of screening results in MTP/LCHAD deficiency. Our report strongly emphasizes the recommendation that patients with suspicious newborn screening results should immediately be referred to specialized centers.

## Abbreviations

HELLP: Hemolysis elevated liver enzymes low platelet count
LCHAD: Long-chain 3-hydroxy acyl CoA dehydrogenase
LCHADD: Long-chain 3-hydroxyacyl-CoA dehydrogenase deficiency
LCKAT: Long-chain 3-ketoacyl-CoA thiolase
MCT: Medium-chain triglycerides
MS/MS: Tandem mass spectrometry
MTP: Mitochondrial trifunctional protein
NBS: Newborn screening
PEG: Percutaneous endoscopic gastrostomy
VLCAD: Very long-chain acyl CoA dehydrogenase
VLCADD: Very long-chain acyl CoA dehydrogenase deficiency
